# Forkhead Transcription Factor Fd3F Cooperates with Rfx to Regulate a Gene Expression Program for Mechanosensory Cilia Specialization

**DOI:** 10.1016/j.devcel.2012.05.010

**Published:** 2012-06-12

**Authors:** Fay G. Newton, Petra I. zur Lage, Somdatta Karak, Daniel J. Moore, Martin C. Göpfert, Andrew P. Jarman

**Affiliations:** 1 Centre for Integrative Physiology, School of Biomedical Sciences, University of Edinburgh, George Square, Edinburgh EH8 9XD, UK; 2 Department of Cellular Neurobiology, University of Göttingen, Burckhardtweg 13, 37077 Göttingen, Germany

## Abstract

Cilia have evolved hugely diverse structures and functions to participate in a wide variety of developmental and physiological processes. Ciliary specialization requires differences in gene expression, but few transcription factors are known to regulate this, and their molecular function is unclear. Here, we show that the *Drosophila* Forkhead box (Fox) gene, *fd3F*, is required for specialization of the mechanosensory cilium of chordotonal (Ch) neurons. *fd3F* regulates genes for Ch-specific axonemal dyneins and TRPV ion channels, which are required for sensory transduction, and retrograde transport genes, which are required to differentiate their distinct motile and sensory ciliary zones. *fd3F* is reminiscent of vertebrate *Foxj1*, a motile cilia regulator, but *fd3F* regulates motility genes as part of a broader sensory regulation program. Fd3F cooperates with the pan-ciliary transcription factor, Rfx, to regulate its targets directly. This illuminates pathways involved in ciliary specialization and the molecular mechanism of transcription factors that regulate them.

## Graphical abstract

**Graphical Abstract:**

**Figure:**
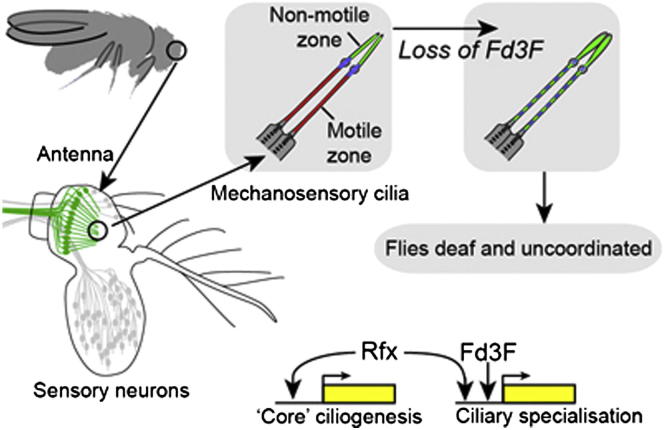

**Highlights:**

► Fd3F is a Fox transcription factor for mechanosensory ciliary specialization ► Regulates genes for ciliary motility, compartmentalization, and sensory transduction ► Fd3F modulates the activity of the pan-ciliogenic transcription factor, Rfx ► Fd3F is likely related to Foxj1, the vertebrate regulator of motile ciliated cells

## Teaser

Cilia are highly diverse. The motile sensory cilia of *Drosophila* mechanosensory neurons provide a good model of ciliary structural and functional specialization. Newton et al. show that Fox and Rfx transcription factors work in concert to activate a gene expression program for ciliary specialization in these neurons.

## Introduction

The cilium commonly constitutes a cell's organelle for environmental sensing in a wide variety of contexts, from developmental signaling pathways (such as Sonic hedgehog) to the specialized receptor processes in sense organs of various sensory modalities (Ishikawa and Marshall, 2011). In other situations, cilia are motile and play many roles connected to fluid movement in the airways, CNS, oviduct, and embryonic node. Despite this structural and functional diversity, cilia share a highly conserved pathway of ciliogenesis, involving basal body docking, axoneme extension, intraflagellar transport (IFT), and ciliary membrane assembly (Silverman and Leroux, 2009). A major question is how this common assembly program is adapted and modified with cell-specific variations to generate cilia diversity (Silverman and Leroux, 2009). It is likely that such specialization requires cell-type-specific gene expression programs, but little is known of the nature of these programs or of the transcription factors that regulate them (Thomas et al., 2010).

In metazoans ciliogenesis broadly depends on regulatory factor X (Rfx) transcription factors (Chu et al., 2010). Their targets are well characterized in functional and bioinformatic studies on *C. elegans* and *Drosophila* (Avidor-Reiss et al., 2004; Efimenko et al., 2005; Laurençon et al., 2007), and include genes required for “core” ciliogenesis processes such as anterograde IFT and membrane assembly. If Rfx is required broadly for ciliogenesis, what regulates cilium specialization? In fact Rfx targets include genes restricted to subtypes of ciliated cell (Efimenko et al., 2005), but it is not clear how it regulates such genes. It is speculated that Rfx cooperates with cell-type-specific factors (Silverman and Leroux, 2009; Thomas et al., 2010), but the nature of this cooperation is uncharacterized.

Few cell-type-specific regulators of ciliogenesis have been characterized. The most well known are members of the FoxJ subfamily. In vertebrates, *Foxj1* has been associated particularly with the differentiation of motile ciliated cell types (Brody et al., 2000; Jacquet et al., 2009; Stubbs et al., 2008; Yu et al., 2008). For instance, *Foxj1* knockout mice have left-right asymmetry and airway defects (Brody et al., 2000). Target gene analyses have shown that *Foxj1* regulates, directly or indirectly, many genes linked to ciliary motility, including axonemal dyneins, but not core ciliogenesis, such as IFT factors (Jacquet et al., 2009; Stubbs et al., 2008; Thomas et al., 2010; Yu et al., 2008). However, very little is known of its molecular mode of action or its relationship with Rfx function.

**Figure 1:**
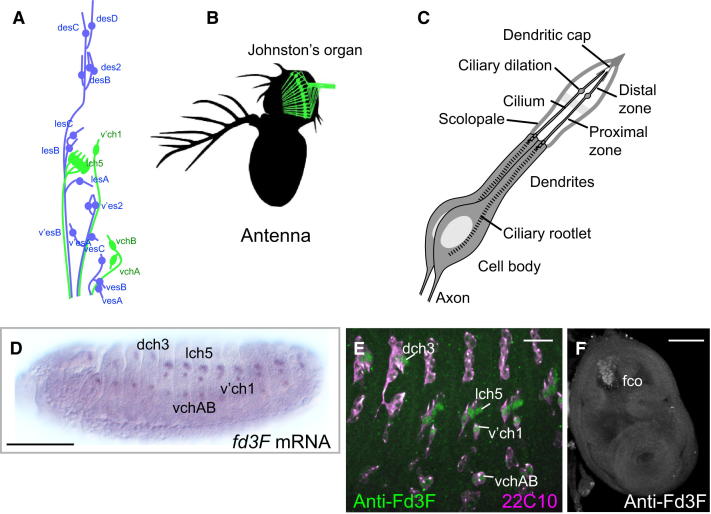
*fd3F* Is Expressed Specifically in Developing Ch Neurons (A) Schematic arrangement of ciliated sensory neurons in an embryonic abdominal segment. Ch neurons, green; ES neurons, blue. (B) Schematic of the array of Ch neurons in the adult antenna that form Johnston's organ. (C) Schematic of a unit Ch organ from Johnston's organ, housing two Ch neurons. (D) *fd3F* mRNA in stage 14 embryos. (E) Fd3F protein localized in a subset of sensory neurons (marked by 22C10) corresponding to Ch neurons (stage 15 embryo). (F) Leg imaginal disc, Fd3F protein in Ch precursor cells of the femoral chordotonal organ (fco). Scale bars, 100 μm (D and F) and 20 μm (E).

In *Drosophila* the only somatic cells with cilia are bipolar sensory neurons, which have specialized ciliary dendrites for sensory reception and transduction (Figures 1A–1C). Different classes of such neurons have ciliary dendrites that have different morphologies, express different sets of receptor molecules, and respond to different sensory modalities. These neurons present a useful model for investigating how ciliary diversity arises. External sensory (ES) neurons have a short connecting cilium leading to a distal sensory process with an irregular core of microtubules. In contrast, proprioceptive and auditory chordotonal (Ch) neurons have a long sensory cilium with a well-defined 9+0 axoneme (Figures 1B and 1C) (Eberl et al., 2000; Kernan et al., 1994). The Ch cilium has several unique specializations. First, its mechanosensory transduction mechanism uniquely involves TRPV (transient receptor potential) channels encoded by *nanchung* (*nan*) and *inactive* (*iav*) (Gong et al., 2004; Kim et al., 2003). Second, it can be motile. In the auditory Ch neurons of Johnston's organ in the antenna (Figure 1B), motility arises from the interplay between transduction channels and adaptation motors (possibly axonemal dyneins). Sensory-induced motility is thought to underlie a mechanical amplification mechanism to increase auditory sensitivity (Göpfert et al., 2005; Göpfert and Robert, 2003). Third, the Ch neuron cilium is divided by a ciliary dilation into functionally distinct proximal (motile) and distal (sensory) zones (Figure 1C) (Lee et al., 2008).

We asked, therefore, what regulates gene expression for Ch neuron-specific ciliary specialization. *Rfx* is expressed in and required for ciliogenesis in both ES and Ch neurons (Dubruille et al., 2002; Laurençon et al., 2007), whereas *Foxj1* homologs are reportedly absent from *Drosophila* (Larroux et al., 2008; Mazet et al., 2003). We recently reported, however, that the Fox gene, *fd3F*, is required for Ch neuron function (Cachero et al., 2011). Here, we show that *fd3F* does not regulate ciliogenesis per se but directly regulates the genes required for aspects of Ch ciliary specialization. It appears that Fd3F cooperates closely with Rfx to regulate this Ch-specific cohort of genes and, therefore, it acts as a cell-type-specific modulator of Rfx target gene specificity. Comparison with *Foxj1* and its target genes suggests that *fd3F* is a highly diverged relative of *Foxj1*.

## Results

### *fd3F* Mutation Results in Uncoordinated Flies with Nonfunctional Ch Neurons

Previously, a transcriptome analysis of embryonic Ch cells revealed that *fd3F* (*CG12632*) was highly enriched in developing Ch neurons, and RNA in situ hybridization and immunostaining showed that *fd3F* is exclusively expressed in Ch cell lineages during embryogenesis (Cachero et al., 2011) (Figures 1D and 1E). In larval imaginal discs, *fd3F* expression was confined to locations of Ch precursors, including the femoral Ch precursors in the leg disc (Figure 1F) and the Ch precursors of Johnston's organ in the antennal disc (data not shown). In summary, *fd3F* expression is unique to developing and differentiating Ch neurons.

Using the fly stock P{EP}*fd3F^EP1198^*, we generated a deletion of 1.4 kb (*fd3F^1^*) that removes approximately 400 bp of the 3′ end of the *fd3F* ORF (see Figure S1A available online) (Cachero et al., 2011). *fd3F* mRNA expression was very strongly reduced in homozygous mutant embryos (Figures S1B and S1C), suggesting that the truncated *fd3F* transcripts are unstable and that the mutation is close to a genetic null. Consistent with this, little difference was observed between the phenotypes of *fd3F^1^* homozygotes and hemizygotes over a deficiency (*fd3F^1^*/*Df(1)ED6716*) in the analyses below.

**Figure 2:**
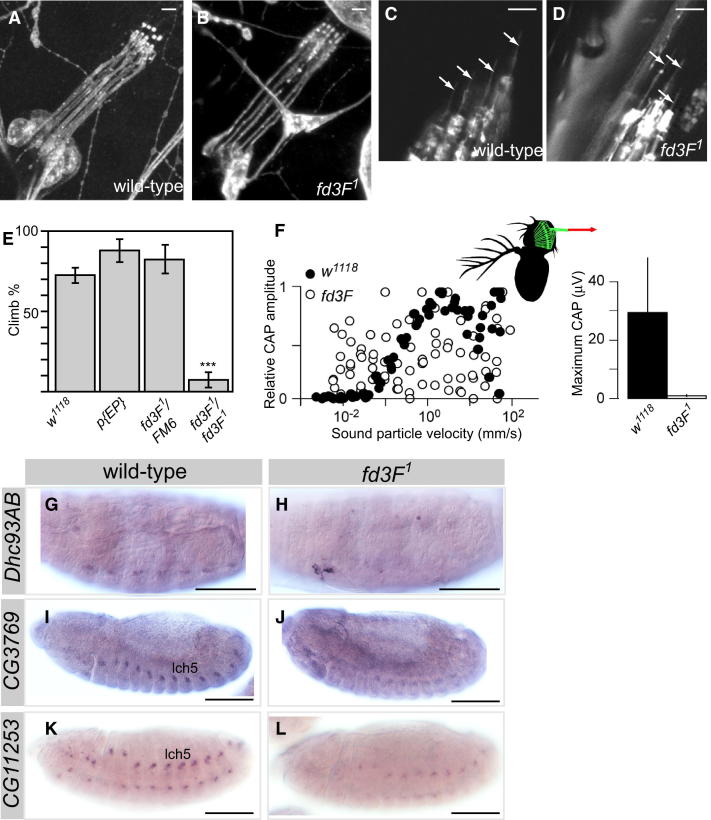
Mutation of *fd3F* Results in Functionally Defective Ch Neurons (A and B) Cell bodies and dendrites of Ch neuron group (lch5) in third-instar larvae stained with anti-HRP. (C and D) Adult femoral Ch organ neurons showing sensory cilia (arrows) labeled with mCD8-GFP (*elav*Gal4;UAS-mCD8-GFP).Scale bars, 5 μm. (E) Climbing assay showing percentage of adult flies climbing above a threshold. Genotypes are wild-type (*w^1118^*), flies homozygous for the P element insert at *fd3F* (p{EP}), *fd3F^1^* heterozygotes (*fd3F^1^*/FM6 balancer), and *fd3F^1^* homozygotes. Error bars are SD. ^∗∗∗^p < 0.0001. (F) Shown on the left are relative CAP amplitudes recorded from the projections of Johnston's organ neurons in the antennal nerve (inset) as a function of the sound particle velocity. Shown on the right are corresponding absolute values of the maximum CAP amplitudes (n = 5 flies per strain). Error bars are SD. (G–L) Genes that show reduced expression in *fd3F* mutant embryos. Wild-type (left) and mutant (right) embryos showing mRNA expression of Ch-expressed genes (named at left) are shown. Expression is either virtually absent (e.g., *Dhc93AB*) or reduced (e.g., *CG11253*). Scale bars, 100 μm. See also Figure S1.

*fd3F^1^* homozygote flies are viable and fertile but are uncoordinated (Cachero et al., 2011), and hence, the adult flies perform very poorly in a climbing assay (Figure 2E). This suggests that Ch neurons are defective in *fd3F* mutants. However, Ch neurons were present in *fd3F^1^* embryos, larvae, and pupae and showed little gross morphological defect (Figures 2A and 2B). When marked by the expression of mCD8-GFP, the Ch sensory cilia were present and grossly normal (Figures 2C and 2D). To assess the functional integrity of Ch neurons in *fd3F* mutants, we examined the activity of the auditory Ch neurons of Johnston's organ. Compound action potentials (CAPs) were recorded in response to auditory stimulus from the antennal nerve of adult flies. Unlike wild-type flies, *fd3F* mutant flies showed no sound-evoked CAPs in the antennal nerve (Figure 2F). These data suggest that *fd3F* regulates aspects of Ch neuronal differentiation that are essential for Ch neuron function.

### *fd3F* Regulates a Subset of Ch-Specific Genes

As we previously reported (Cachero et al., 2011), *fd3F* is required for the expression of the genes *nan* and *iav*, which encode subunits of a TRPV cation channel uniquely localized to Ch neuron cilia (Gong et al., 2004; Kim et al., 2003). To identify other regulatory targets of *fd3F*, we began by examining candidate Ch-expressed genes identified previously in our transcriptome analysis (Cachero et al., 2011). Of 16 genes tested initially, 6 showed reduced expression in *fd3F* mutant embryos (Figures 2G, 2H, and S1D; Table S1). Thus, *fd3F* does not regulate all Ch neuron-specific genes. Instead, the nature of the target genes suggests that *fd3F* regulates specific processes in Ch sensory specialization.

### *fd3F* Regulates Genes Required for Ch Ciliary Motility

**Figure 3:**
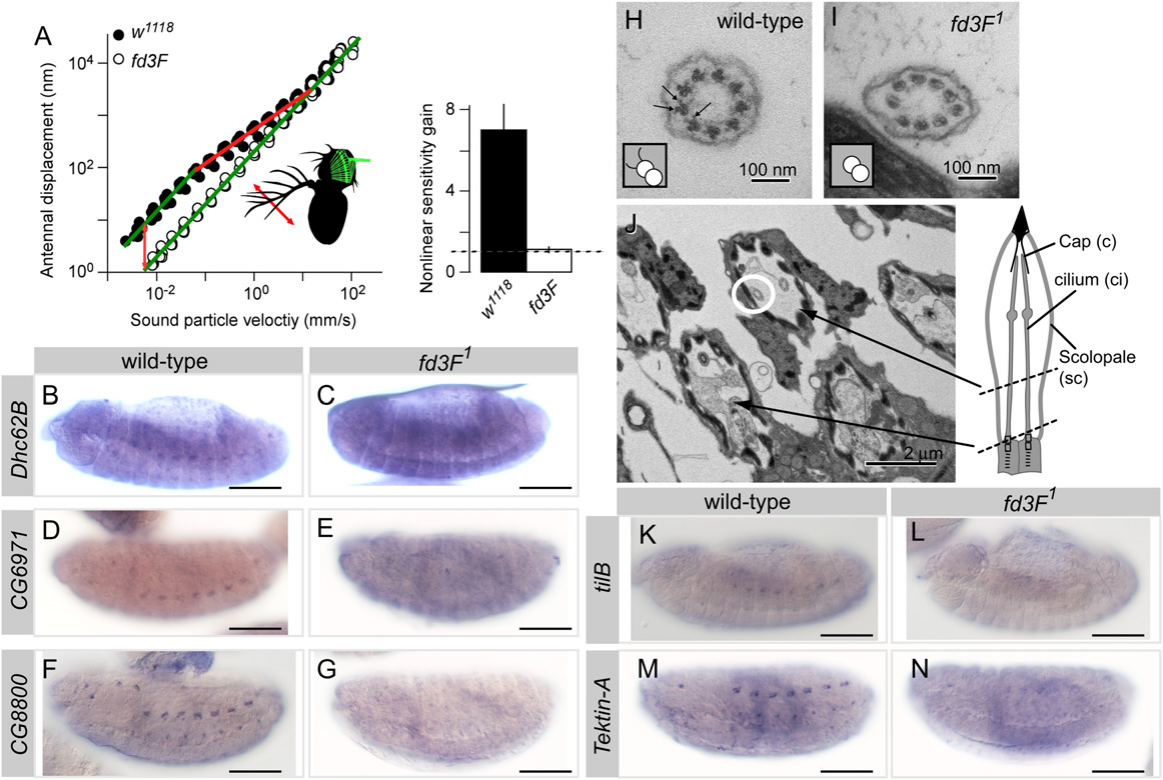
*fd3F* Regulates Genes Required for Ch Ciliary Motility (A) Shown on the left is a tone-evoked antennal displacement amplitude as a function of the sound particle velocity at the arista tip (inset, red arrow). Green lines indicate linearity, red lines nonlinearity. The orange arrows highlight the nonlinear sensitivity gain. Shown on the right is nonlinear sensitivity gain. A gain of one (hatched horizontal line) signals the absence of amplification. Error bars are SD. (B–G) mRNA expression of axonemal dynein genes in wild-type and *fd3F* mutant embryos. (B, D, and F) In wild-type embryos, these genes are Ch specific but rather weakly expressed (Ch cell transcriptome analysis supports this, Table S1). (C, E, and G) Expression is strongly reduced in *fd3F* mutant embryos. (H and I) TEM of Ch cilium cross-sections from adult antennae (Johnston's organ), showing the nine axonemal microtubule doublets. Axonemal dynein arms can be seen in wild-type (H, arrows), but not in *fd3F* mutant (I). Insets show this phenotype schematically. (J) Lower-magnification view of Ch units with the cilium in (I) ringed. The schematic shows the approximate location of the cross-sections. (K–N) Expression of genes required for axonemal dynein assembly or function shows Ch-specific (albeit weak) expression (K and M) that is strongly reduced in *fd3F* mutants (L and N). Scale bars, 100 μm. See also Table S1.

The *fd3F* target, *Dhc93AB* (Figures 2G and 2H), encodes an axonemal outer-arm dynein heavy chain (homolog of human DNAH9/11). Axonemal dyneins form a large family of multisubunit motors that are responsible for cilium motility. In *Drosophila* embryos, Ch neurons bear the only cilia with axonemal dynein arms, and correspondingly, *Dhc93AB* is expressed in a Ch-specific pattern. In Ch neurons of Johnston's organ, nonlinear mechanical amplification arises from the motility of Ch cilia (Göpfert et al., 2005; Göpfert and Robert, 2003; Nadrowski et al., 2008). Axonemal dyneins are good candidates to contribute to this process. Like vertebrate hair cells, Ch neurons use mechanical amplification to augment the tiny sound-induced vibrations that they transduce. Active amplification can be tested by examining the mechanical response of the antennal sound receiver to tones of different intensities (Göpfert et al., 2006). In wild-type flies this response displays a compressive nonlinearity that enhances sensitivity about 10-fold when sound is faint (Figure 3A). This nonlinearity was completely absent in *fd3F* mutant flies (Figures 3A and 3B), demonstrating that Ch cilium motility is lost.

Besides *Dhc93AB*, a number of other axonemal dynein components are present in transcriptome of differentiating embryonic Ch neurons (Cachero et al., 2011) (Table S1), and several of these were confirmed to have Ch-specific expression by in situ hybridization (Figures 3B, 3D, and 3F; Table S1). This suggests that axonemal dyneins are important in embryonic (proprioceptive) as well as antennal (auditory) Ch neurons. Of these genes, almost all were strongly reduced or absent in *fd3F* mutant embryos, including inner-arm dyneins *Dhc16F* (human DNAH6) and *Dhc62B* (DNAH3/7), axonemal dynein light chains *CG8800* (DNAL1) and *CG34192* (DNALRB2), light-intermediate chain *CG6971* (DNALI1), and intermediate chain *CG13930* (IC138, WDR78) (Figures 3B–3G; Table S1). Together, these observations suggest that *fd3F* regulates axonemal dynein genes in general. To check that this also pertains to the Johnston's organ, Ch neuron ultrastructure in the antenna was examined by transmission electron microscopy (TEM). In transverse sections of mutant ciliary dendrites, normal axoneme arrangement and ultrastructure were observed, but dynein outer and inner arms were missing from the proximal cilium (Figures 3H–3J). Loss of dyneins with consequent loss of motility can account for the loss of mechanical amplification by Johnston's organ neurons in *fd3F* mutants, although *fd3F* might also regulate genes of the sensory transduction apparatus that are required for the process (Nadrowski et al., 2008).

Several genes have been implicated in axonemal dynein assembly. *tilB* is required for transport or assembly of axonemal dyneins (Kavlie et al., 2010), and *CG14905* is similar to *Chlamydomonas ODA-1*, which is required for axonemal dynein assembly (Kamiya, 1988). Ch neuron expression of both genes was reduced in *fd3F* mutant embryos (Figures 3K and 3L; data not shown). Tektins are required for correct motility of cilia, probably by participating in dynein inner-arm assembly or attachment (Amos, 2008). We found that *Tektin-A* (*TEKT4* ortholog) is expressed exclusively in Ch neurons and that its expression is strongly reduced in *fd3F* mutants (Figures 3M and 3N). Thus, *fd3F* regulates genes required for several aspects of dynein arm formation.

### Fd3F Regulates Retrograde Transport Genes to Form Functionally Specialized Ciliary Zones

**Figure 4:**
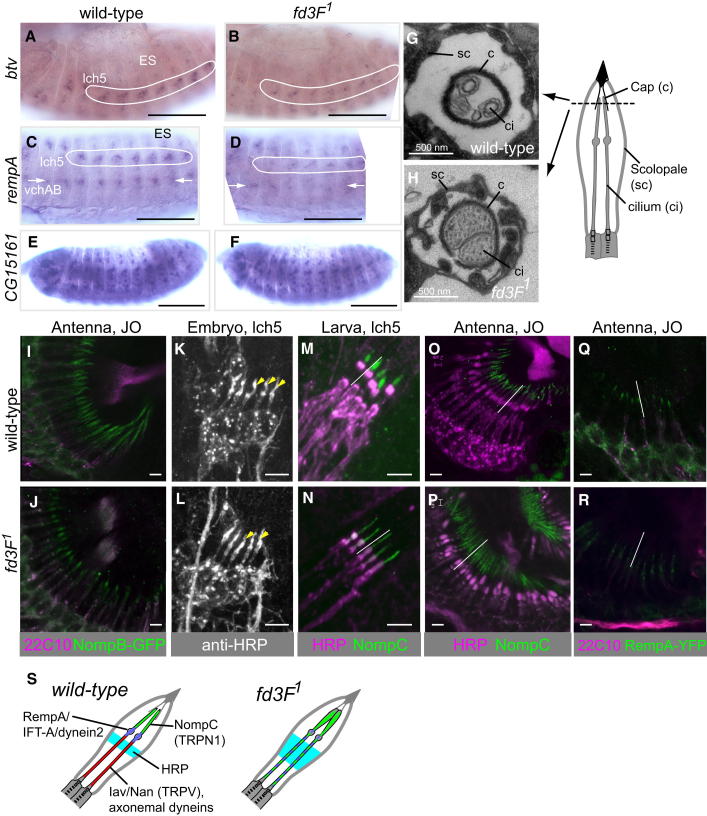
*fd3F* Regulates Genes Required for Ciliary Compartmentalization (A–D) mRNA expression of retrograde IFT-related genes in wild-type and *fd3F* mutant embryos. These genes show a Ch-enriched expression pattern of which the Ch neuron component (ringed and marked with arrows) is reduced in *fd3F* mutants. (E and F) The expression of an anterograde IFT gene, *CG15161* (in a pattern consistent with expression in all ciliated sensory neurons), is unaffected in *fd3F* mutant embryos. Scale bars, 20 μm. (G and H) TEM cross-section of the tip of a Ch organ unit from adult antennal Johnston's organ. The Ch cilium tips (ci) are housed within the dendritic cap (c) (schematic shows the location of the cross-section). As shown in (G), the cilium tips are enlarged to fill the cap in the *fd3F* mutant antenna (H). (I and J) NompB-GFP fusion protein accumulates at the tips of Johnston's organ Ch neurons in *fd3F* mutant pupal antennae (cell bodies counterstained with 22C10). (K) In wild-type embryonic Ch neurons, anti-HRP marks the Ch cilia including the ciliary dilation (arrowheads). (L) The dilation is not apparent in Ch cilia from *fd3F* mutant embryos (arrowheads indicate the expected location of the dilation). (M–P) NompC protein is localized to the distal Ch cilia in wild-type neurons (M and O) but becomes dispersed along the cilium in *fd3F* mutant neurons (N and P). Lines indicate the approximate extent of the cilium. (Q) RempA-YFP protein is localized in the vicinity of the ciliary dilation in wild-type Ch cilia. (R) In *fd3F* mutant cilia, RempA-YFP protein becomes dispersed. (S) Schematic summary of protein localizations in wild-type and mutant Ch neuron cilia. Scale bars, 4 μm (I, J, and O–R) and 5 μm (K–N). See also Table S1.

The *fd3F* target, *CG3769* (Figures 2I and 2J), encodes the ortholog of cytoplasmic dynein 2 light-intermediate chain (DYN2LIC). Cytoplasmic dynein 2 is the motor for retrograde IFT during ciliogenesis, and dynein 2 heavy chain (DYN2HC), encoded by *beethoven* (*btv*), is required for Johnston's organ function (Eberl et al., 2000). Unlike axonemal dyneins, *btv* expression is not strictly confined to Ch neurons: it is expressed preferentially in Ch neurons but also at a lower level in ES neurons (the so-called *Ch-enriched* pattern; Cachero et al., 2011) (Figure 4A). This is consistent with retrograde transport being required for ciliogenesis in both sensory neuron subtypes. Like *CG3769*, *btv* mRNA is reduced in the Ch neurons of *fd3F* mutant embryos (Figure 4B). However, some expression remains in Ch neurons, and ES neuron expression appears unaffected.

Retrograde transport also requires proteins of the IFT-A complex, and we previously found that IFT-A genes are also expressed in the Ch-enriched pattern (Cachero et al., 2011). In *fd3F* mutant embryos, *rempA* (IFT140) and *Oseg6* (IFT144) mRNAs were both reduced in Ch neurons (Figures 4C and 4D; data not shown). *Oseg1* was also reduced in whole embryos as shown by RT-PCR (data not shown). In contrast to retrograde transport genes, the anterograde IFT-B genes, *nompB* (IFT88) and *CG15161* (IFT46), were not affected in *fd3F* mutants (Figures 4E and 4F; data not shown).

In *Chlamydomonas* and other models, disruption of retrograde IFT causes a buildup of proteins at the tip of the cilium, which then becomes swollen (Pazour et al., 1998). This phenotype was present in *fd3F* mutant Johnston's organ dendrites: in transverse sections the tips of the cilia fill the dendritic cap (Figures 4G and 4H). However, the cilia did not appear truncated, as is seen in *rempA* null mutants (Lee et al., 2008), consistent with only a partial loss of retrograde IFT. A second feature of retrograde transport mutants is the accumulation of IFT-B particles in the ciliary tips (Hou et al., 2004). We observed a moderate accumulation of NompB-GFP fusion protein in the tips of *fd3F* mutant cilia (Figures 4I and 4J).

If *fd3F* is responsible for elevated expression of retrograde transport genes in Ch neurons relative to ES neurons, this implies that increased expression has a Ch neuron-specific function. Indeed, although retrograde transport is required for ciliogenesis generally, *rempA* has an additional Ch-specific role of creating and maintaining the distinct proximal motile and distal nonmotile ciliary zones, separated by the ciliary dilation (Lee et al., 2008) (Figure 1A). These zones differ in ion channel composition, with Nan and Iav (TRPV) localized to the proximal zone, whereas the distal zone contains the candidate force-responsive ion channel, NompC (TRPN1 homolog) (Cheng et al., 2010; Liang et al., 2011) (Figures 4M and 4O). In *fd3F* mutant embryos, defective ciliary dilation staining was observed similar to that reported for *btv* mutants (Eberl et al., 2000) (Figures 4K and 4L). We investigated whether this reflects a disruption of ciliary compartmentalization. As noted above, proteins known to be restricted to the proximal zone (axonemal dyneins, Nan, Iav) are themselves *fd3F* targets, and so their localization cannot readily be analyzed. However, distally localized NompC protein could be examined because its transcription is independent of *fd3F* (data not shown). In *fd3F* mutant larvae and adult antennae, NompC protein was not restricted distally but was instead spread along the length of the cilium, suggesting a disruption of ciliary compartmentalization (Figures 4M–4P). Consistent with this, the axonemal ultrastructure of the mutant proximal cilium appears to resemble that of the distal cilium (a dense core is seen in only one microtubule of the doublets) (Figure 3I; data not shown). The RempA-YFP protein itself normally concentrates in the region of the ciliary dilation (Lee et al., 2008) (Figure 4Q). However, in *fd3F* mutant neurons, RempA-YFP was dispersed along the proximal cilium (Figure 4R). In conclusion, *fd3F* not only regulates genes whose products are localized to the proximal motor zone (axonemal dyneins, Iav, Nan) but also upregulates genes required to form and maintain the distinct zones (cytoplasmic dynein 2, IFT-A components) (Figure 4S).

### Direct Regulation of *nan* and *iav* by Fd3F

**Figure 5:**
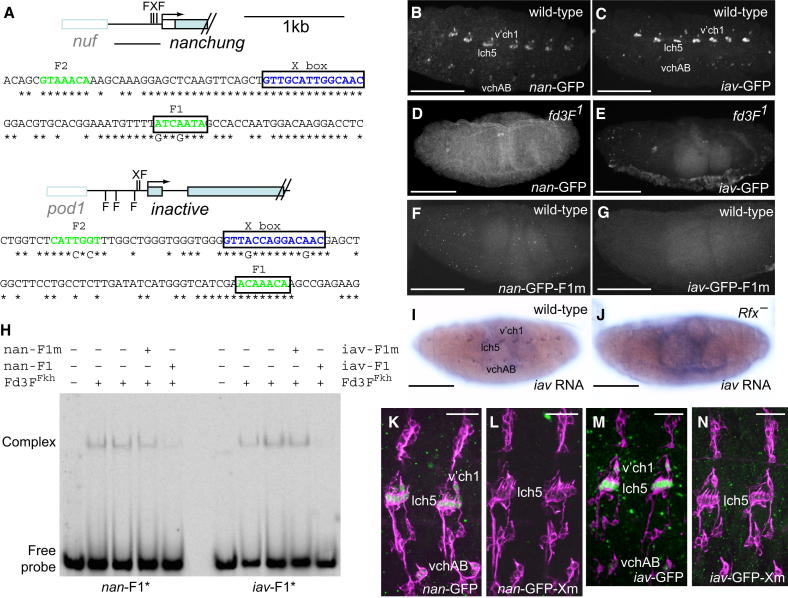
Fd3F Directly Regulates *nan* and *iav* with Rfx (A) Schematics of the *nan* and *iav* genes showing locations of Fox motifs (F) and X boxes (X) in their upstream regions. The sequences surrounding the X boxes are shown, with asterisks representing identity in *D. pseudoobscura*. (B and C) Stage 16 embryos showing expression of GFP in Ch neurons for *nan-*GFP (B) and *iav*-GFP (C) reporter genes. (D and E) Expression of both reporter genes is lost in *fd3F* mutant embryos. (F and G) Expression of both reporter genes is abolished upon mutation of a single Fox motif (F1) in either upstream region. (H) Gel mobility shift assay with oligonucleotide probes (^∗^) containing the *nan*-F1 or *iav*-F1 Fox motifs and purified Fd3F forkhead domain polypeptide (Fd3F^Fkh^). The DNA-Fd3F^Fkh^ complexes are indicated. Addition of 100-fold excess of cold competitor oligonucleotide (*nan*-F1, *iav*-F1) reduces complex formation, but when the Fox motif is mutated (*nan*-F1m, *iav*-F1m), the cold competitor has no effect on complex formation. (I and J) Embryonic expression of *iav* in Ch neurons (I) is lost in *Rfx^49^* homozygous mutant embryo (J) (embryos stained in parallel). (K–N) Two abdominal segments from stage 16 embryos stained with anti-GFP (green) and 22C10 (magenta). Ch neuron expression of *nan*-GFP (K) and *iav*-GFP (M) reporter genes is abolished when the upstream X box is mutated (L and N). Scale bars, 100 μm (B–J) and 20 μm (K–M). See also Table S1.

We examined the *cis* regulation of *nan* and *iav*. For *nan* a 557 bp upstream fragment (−561 to −4, relative to ATG) drives GFP reporter expression specifically in embryonic Ch neurons (*nan*-GFP) (Kim et al., 2003) (Figures 5A and 5B). In *fd3F^1^* mutant embryos, *nan*-GFP expression was abolished (Figure 5D), showing that this enhancer is responsive to *fd3F*. Similarly, for *iav* a 597 bp upstream fragment was able to drive Ch-specific GFP expression in an *fd3F*-dependent manner (Figures 5A, 5C, and 5E). Fox factors of several subfamilies bind to a common sequence in vitro, RYMAAYA (Benayoun et al., 2011). The *nan* and *iav* enhancers contain several such motifs. Mutation of the Fox motifs closest to the *iav* and *nan* translational start sites (*iav*-F1 and *nan*-F1) abolished *iav-GFP* and *nan-GFP* reporter gene expression (Figures 5F and 5G). In contrast, mutation of Fox motifs slightly further upstream (F2 in Figure 5A) had no effect on reporter gene expression (data not shown). To test whether Fd3F can bind to these F1 motifs, the DNA binding Forkhead domain of Fd3F (Fd3F^Fkd^) was expressed as a GST fusion protein and isolated from bacterial cells. When used in an in vitro gel mobility shift assay, GST-Fd3F^Fkd^ was able to bind specifically to the F1 sites of both *nan* and *iav* (Figure 5H).

### Coregulation of Ciliary Specialization Genes by Fd3F and Rfx Transcription Factors

Typically, Rfx factors regulate target genes via a single upstream binding site (X box) (Efimenko et al., 2005; Laurençon et al., 2007). The *iav* and *nan* enhancer sequences contain an X box motif similar to that determined biochemically for human Rfx1 (GTNRCCN{0-3}RGYAAC; Emery et al., 1996), very close to the functional Fd3F binding sites (Figure 5A). This raises the possibility that although Rfx is a pan-ciliary transcription factor required for both Ch and ES neurons (Dubruille et al., 2002), it contributes to the regulation of Ch-specific Fd3F target genes. Indeed, both *nan* and *iav* expression were strongly reduced in *Rfx* mutant embryos (Figures 5I and 5J; data not shown). In both cases mutation of the X box in the reporter constructs largely abolished enhancer activity (Figures 5K–5N). We therefore conclude that an X box/Fox motif combination is required for *nan* and *iav* regulation in Ch neurons and that most likely these sites are bound by Rfx and Fd3F. In support of the conclusion that these transcription factors are coregulators, we found no evidence of a regulatory hierarchy between *Rfx* and *fd3F*: in embryos we found that *fd3F* transcription does not depend on *Rfx* function and vice versa (data not shown).

**Figure 6:**
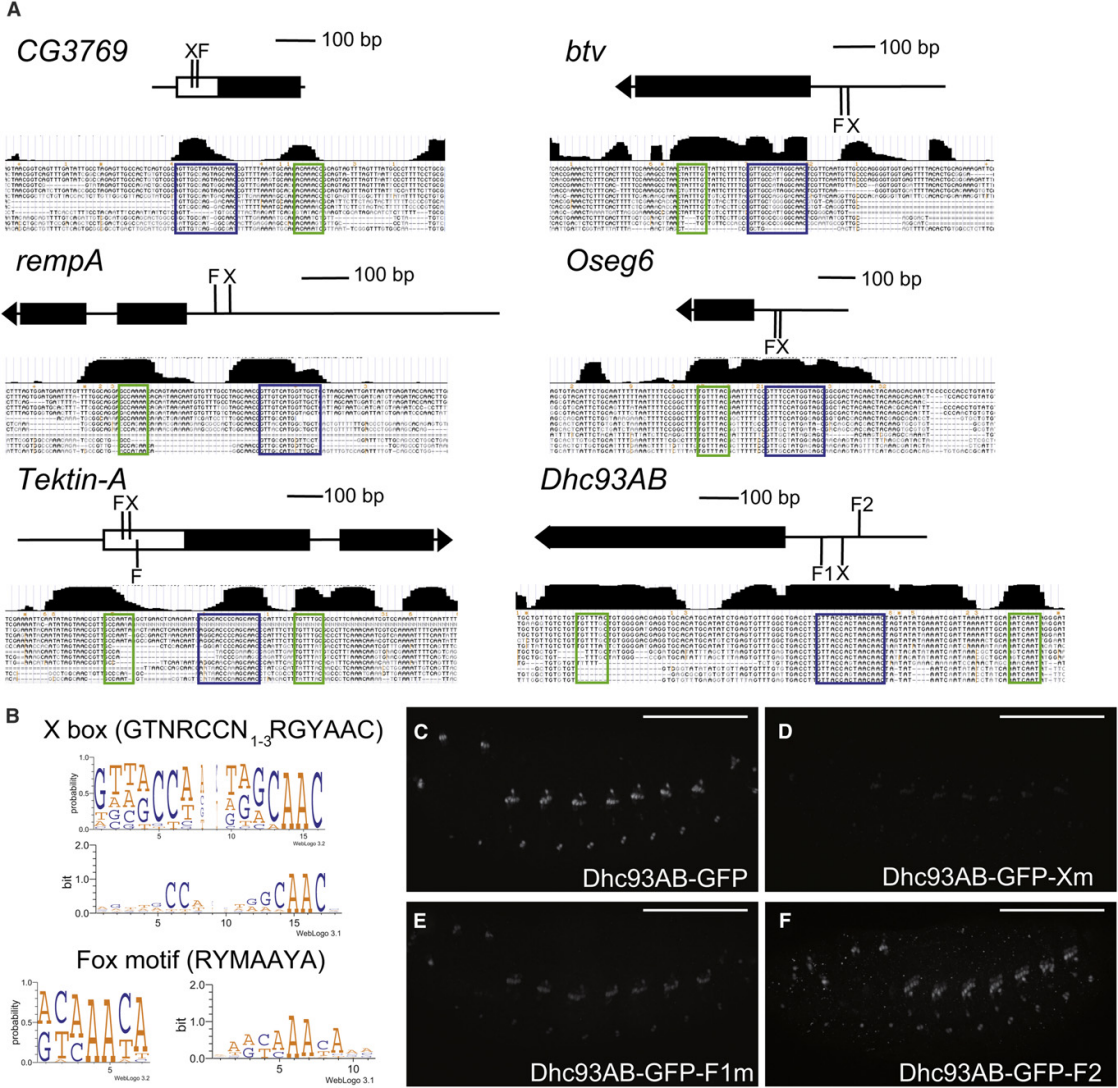
Promoter Regions of Fd3F/Rfx Target Genes Contain a Paired Fox Motif/X Box Combination (A) Schematics of example gene regions (first exons and upstream regions only) with locations of X box/Fox motif pair. Below each is a screenshot from the UCSC genome browser (http://genome.ucsc.edu/) representing the sequence alignment of 12 *Drosophila* species and showing degree of identity as a histogram. X boxes and Fox motifs are outlined in blue and green, respectively. (B) Sequence logos (http://weblogo.threeplusone.com) summarizing the alignment of X boxes (left) and Fox motifs (right) from all identified *fd3F* target genes (see Table S2). The representations of information content (bits) emphasize the fact that the X boxes often comprise one strong half-site and a more degenerate one. (C–F) Embryos containing a *Dhc93AB-GFP* reporter gene. (C) Stage 16 embryo showing specific expression of *Dhc93AB-GFP* in Ch neurons. (D) Mutation of a proximal X box strongly reduces expression. (E) Mutation of Fox motif F1 reduces expression. (F) Mutation of Fox motif F2 has no discernable effect. Scale bars, 100 μm. See also Table S2.

When we examined a selection of other *fd3F* target genes (including *Dhc93AB*, *rempA*), we found that their expression was also strongly reduced in *Rfx* mutant embryos (Table S1). Moreover, we observed that almost all *fd3F* target genes contain a closely spaced combination of conserved Fox and X box motifs, usually within 50 bp of the transcriptional or translational start site (Figure 6A; Table S2). Some of these genes were suspected Rfx direct targets from other bioinformatic analyses (Avidor-Reiss et al., 2004; Laurençon et al., 2007) (Table S2). However, most of these genes were not previously predicted to be Rfx targets, partly due to the fact that the X box motif often does not conform completely to the palindromic consensus sequence previously considered for *Drosophila* targets: GTTGCCATGGCAAC (Avidor-Reiss et al., 2004); GYTRYYN(1-3)RRHRAC (Laurençon et al., 2007) (Figure 6B). The juxtaposition of Fox motifs and X boxes suggests that Fd3F and Rfx are coregulators of a subset of Ch ciliary genes. Subsequent to the bioinformatic analysis, we confirmed that the regulation of *Dhc93AB* indeed mirrors that of *nan* and *iav*. An 820 bp promoter fragment supports Ch neuron-specific reporter gene expression, and mutation of the proximal-most X box and Fox motifs reduced this expression. However, in this case expression was not completely abolished, possibly due to redundancy with other binding sites that are present in this longer enhancer (Figures 6C–6F).

### Target Genes Are Ectopically Activated upon Fd3F Misexpression

**Figure 7:**
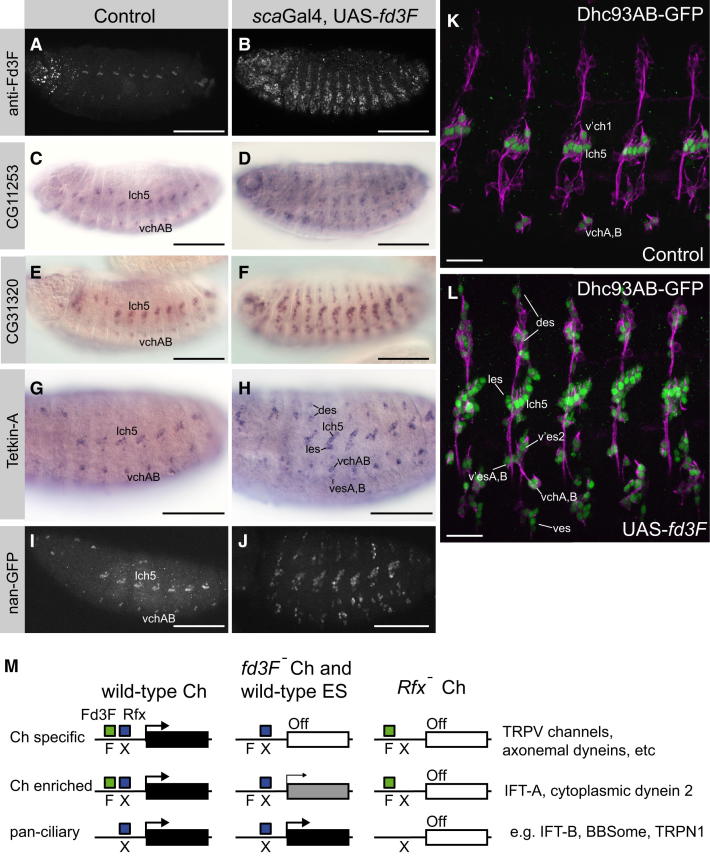
Ectopic Activation of Target Genes by Fd3F Misexpression (A and B) Expression of Fd3F in embryos from control (UAS-*fd3F*, no driver) and misexpressing (*scaGal4*, UAS-*fd3F*) lines. (C–H) Expression of *fd3F* target genes. In (H), some of the ES neurons exhibiting ectopic expression of *Tektin-A* are indicated (cf. Figure 1A). (I and J) Expression of *nan-GFP* reporter gene. (K and L) Expression of *Dhc93AB-GFP* reporter gene. ES neurons exhibiting ectopic expression are labeled in (L). (M) Summary of target gene regulation by *fd3F* and *Rfx* is shown. In wild-type Ch neurons, Fd3F and Rfx cooperate to regulate a subset of Ch-specific and Ch-enriched genes required for ciliary specialization. In ES neurons, and in *fd3F* mutant Ch neurons, Rfx does not activate the Ch-specific genes but is able to activate Ch-enriched genes at a low level for “basal” retrograde transport. In Rfx mutants, Fd3F is unable to activate these genes alone. pan-ciliary genes are regulated by Rfx in Ch and ES cells. Note that in addition to these interactions, it is possible that ES neurons express their own ciliary specialization regulators, so that *fd3F* mutant Ch cilia do not “revert” to an ES cilium state. Scale bars, 100 μm (A–J) and 20 μm (K and L).

Using flies transgenic for an inducible UAS-*fd3F* construct, we ectopically expressed Fd3F in embryonic sensory precursor cells using a *scaGal4* neuroectodermal driver line (Figures 7A and 7B). This induced the misexpression of several *fd3F* target genes in ES neurons, including *CG8800* (*DNAL1*), *Tektin-A*, *CG11253*, and *CG31320* (Figures 7C–7H; data not shown). In addition the *nan*, *iav*, and *Dhc93AB* GFP reporter gene constructs were all ectopically expressed upon *fd3F* misexpression (Figures 7I–7L; data not shown). Thus, *fd3F* is sufficient to activate aspects of Ch-specific gene expression in other sensory neurons. Interestingly, although *scaGal4* drives expression widely in neuroectodermal cells, misexpression of target genes appears to be confined to ES neurons (Figures 7K and 7L). This is consistent with the idea that Fd3F function is limited to cells expressing its coregulator, Rfx.

## Discussion

Fd3F is a cell-type-specific transcriptional regulator of ciliary sensory specialization. It is exclusively expressed in mechanosensory Ch neurons where it regulates aspects of Ch neuron ciliogenesis and ciliary function. Fd3F is not a Ch neuron identity factor or “master regulator” of Ch neuron differentiation: neural differentiation and ciliogenesis occur largely normally in *fd3F* mutant Ch neurons as attested by general morphology, and many Ch-specific genes are *fd3F* independent. Instead, *fd3F* regulates a program of gene expression for mechanosensory specialization that is unique to Ch neuron cilia and is absolutely required for their response to sensory stimulation. Specifically, *fd3F* targets are concerned with the structurally and functionally distinct ciliary zones of Ch neuron dendrites: *fd3F* regulates genes for both the machinery for construction and delineation of the zones (retrograde transport) and the proteins that populate the specialized proximal motor zone (axonemal dyneins, tektin, TRPV proteins). *fd3F* mutation has two consequences: ciliary motility of JO neurons is lost (reflected by loss of mechanical amplification), and sensory transduction is lost (reflected by loss of electrical response to stimulus). Effects on sensory transduction are both direct (loss of TRPV expression) and indirect (disrupted localization of candidate force-gated channel, TRPN1). Loss of axonemal dyneins might underlie the loss of motility directly, or their role may be indirect through their potential function as adaptation motors in sensory transduction (Nadrowski et al., 2008).

Mutation of *fd3F* does not lead to transformation of Ch cilium morphology to that expected for ES neurons—only motility and compartmentalization-related specializations are lost. Similarly, *fd3F* misexpression does not convert ES cilia to a Ch cilium morphology (unpublished data). We suggest that ES and Ch neuron cilia are both specialized derivatives of a default “nonspecialized” cilium structure, such that loss of *fd3F* results only in loss of specific Ch ciliary specializations. There may be other aspects of specialization regulated by other factors in both Ch neurons and other sensory neurons.

Consideration of *fd3F* target genes illuminates the outstanding question of how ciliogenesis pathways are modulated to produce specialized cilia. One might surmise that ciliary specialization requires subtype-restricted gene products. This is true to some extent, as exemplified by the TRPV proteins and axonemal dyneins. However, our findings suggest that quantitative differences in gene expression between sensory neuron subtypes are also important for ciliary specialization. Compartmentalization of Ch cilia into specialized motile and sensory zones requires retrograde transport proteins (Eberl et al., 2000; Lee et al., 2008). Our results suggest that a “basal” level of retrograde transport is sufficient for ciliogenesis in all sensory neurons (as is present in ES neurons and *fd3F* mutant Ch neurons), but compartmentalization of the Ch cilium requires a higher level of activity, as is provided by *fd3F* regulation of the genes involved. Thus, a quantitative difference in gene expression programs might underlie a qualitative feature of Ch ciliary specialization. This can be seen as a variation of the idea that differences in IFT activity might contribute to ciliary specialization (Silverman and Leroux, 2009).

One model for transcriptional regulation of ciliary specialization is that *Rfx* is required for pan-ciliary gene expression, whereas other more restricted transcription factors regulate subtype-specific gene expression (Silverman and Leroux, 2009). However, some targets of *Rfx* are expressed only in subsets of ciliated cells (Efimenko et al., 2005), raising the question of how a pan-ciliary factor can be responsible for subtype-specific gene expression (Silverman and Leroux, 2009; Wang et al., 2010). *Rfx* is required for all ciliated neurons in *Drosophila*, but its target gene specificity is modulated in Ch neurons by *fd3F* (Figure 7M). This suggests a general mechanism in which Rfx cooperates with cell-type-specific transcription factors to regulate the genes required for cilia diversification. Most *fd3F/Rfx* targets have a conserved X box/Fox motif combination, demonstrating that specificity factors (in this case Fd3F) may act in very close molecular cooperation with Rfx, perhaps entailing cooperative binding. Conversely, most if not all *fd3F* target genes are also *Rfx* dependent, such that Rfx appears to be an obligate cofactor of Fd3F. Interestingly, the X boxes associated with *fd3F* targets often do not conform to the classic palindromic Rfx binding site (e.g., GTTGCCATGGCAAC; Avidor-Reiss et al., 2004) but, instead, show a strong match in only one half-site (RGYAAC). Half-sites and modified (nonpalindromic) X box sites have been noted for a variety of other cilia genes and might be especially associated with cell-type-restricted targets (Efimenko et al., 2005; Piasecki et al., 2010).

Interestingly, the same X box/Fox site combination is shared by both Ch-specific and Ch-enriched targets. Therefore, Fd3F acts as an obligatory cofactor of Rfx for Ch-specific genes, but for Ch-enriched genes it only enhances a basal level of *Rfx*-dependent regulation that is already existent in Ch and ES neurons (Figure 7M). To extend our model above, *Rfx* regulates low-level retrograde transport activity sufficient for its “basal” ciliogenesis role, whereas *fd3F/Rfx* regulate the higher activity in Ch neurons that is required for ciliary compartmentalization.

In being required for Ch cilium motility, *fd3F* has a strikingly similar role to vertebrate *Foxj1* genes, albeit that *Foxj1* mutation has wider phenotypic consequences due to the many roles performed by motile cilia in vertebrates. Is *fd3F* related to *Foxj1*? The FoxJ subfamily is ancient, but bioinformatic analyses previously detected no *Drosophila* or *C. elegans* orthologs, suggesting that FoxJ genes have been lost from ecdysozoans (Mazet et al., 2003). Conversely, such analyses also failed to assign *fd3F* to any Fox subfamily (Lee and Frasch, 2004). However, a recent comprehensive analysis of mosquito Fox genes tentatively placed *fd3F* and its mosquito equivalent in the FoxJ subfamily (Hansen et al., 2007). Although the sequence evidence is equivocal, we suggest that *fd3F* is a highly diverged representative of the FoxJ subfamily.

Support for this relationship comes from consideration of target genes. Target gene analyses have indicated many candidate direct or indirect targets for *Foxj1* in mouse and *Xenopus* (Jacquet et al., 2009; Stubbs et al., 2008). Several *fd3F* target genes are homologs of *Foxj1*-dependent genes in multiple vertebrate species (Thomas et al., 2010) (Table S1), including *Dhc93AB* (*DNAH9* in human), *CG9313* (*WDR66*), *tektin-A* (*TEKT4*), *CG6971* (*DNALI1*), *CG13930* (*WDR78*), *Dhc62B* (*DNAH3*), *Dhc16F* (*DNAH6*), *CG10064* (*WDR16*), and *CG34192* (*DNALRB2*). The shared targets are all directly concerned with motility, whereas retrograde transport genes are not *Foxj1* targets in vertebrates. This suggests that regulation of axonemal motor genes is an ancestral function of *Foxj1*/*fd3F*, whereas regulation of retrograde transport was acquired later in the *Drosophila* lineage, coinciding with the emergence of distinct ciliary zones in the evolution of Ch neurons. Other *fd3F* target genes have no known function: we suggest they provide a potential source of new ciliary motility genes.

Cilia are classically classified as either being sensory (primary) with a 9+0 microtubule axoneme or propulsive (motile) with a 9+2 axoneme. Ch cilia are 9+0 sensory cilia with a limited set of motility-related features that are intimately linked to the Ch sensory transduction mechanism. These cilia therefore differ greatly from most propulsive cilia. Several *Foxj1*-dependent cell types also bear 9+0 motile cilia somewhat reminiscent of those on Ch neurons. These include the mouse embryonic node, which is required for left-right asymmetry (Brody et al., 2000), related cells in *Xenopus* and zebrafish (e.g., Kupffer's vesicle), and long 9+0 cilia of the neural tube floor plate in zebrafish (Stubbs et al., 2008; Yu et al., 2008).

In vertebrates, *Rfx3* is required in many ciliated cells that also require *Foxj1*, including the mouse embryonic node, CNS ependymal cells (Baas et al., 2006), and chick neural tube floor plate (Cruz et al., 2010). Moreover, in the embryonic node *Rfx3* regulates genes involved not only with ciliogenesis but also with cilium mobility (Bonnafe et al., 2004), and some *Foxj1*-dependent genes are also affected in *Rfx3* mutants, including *Dnahc9*, the ortholog of *Dhc93AB* (El Zein et al., 2009). We suggest that *Rfx3/Foxj1* may work in combination to regulate directly a subset of ciliary targets in the way we have identified for *Rfx/fd3F*.

## Experimental Procedures

### Fly Stocks

Flies were maintained on standard media at 25°C. We used *w^1118^* as wild-type control. The stocks *elavGal4*, UAS-mCD8-GFP, *p{EP}fd3F^EP1198^*, *Df(1)ED6716, ec^1^* were obtained from the Bloomington Stock Center (Indiana University, Bloomington, IN, USA). Other stocks used were *Rfx^49^* (Dubruille et al., 2002), *rempA*-YFP (Lee et al., 2008), and *nompB*-GFP (Han et al., 2003).

### Immunohistochemistry

Primary antibodies used were mAb-22C10 (1:200), RbAb-HRP (1:500), RbAb-GFP (1:500) (Molecular Probes), and mAb-NompC (Liang et al., 2011). For generating the Fd3F antibody, synthetic peptides matching the C-terminal region of Fd3F were injected into two rabbits to produce anti-Fd3F antisera (CovalAb/Eurogentec). The serum was purified using Melon Gel IgG Spin Purification Kit (Thermo Scientific) and was used at 1:100. Secondary antibodies were from Molecular Probes. mRNA in situ hybridizations to whole embryos were by standard digoxigenin method. Gene fragments were amplified from genomic DNA by PCR (oligonucleotides shown in Supplemental Experimental Procedures) and digoxigenin-labeled RNA probes prepared using T7 RNA polymerase. For bright-field microscopy, embryos were observed using an Olympus AX70 microscope and captured with a DP50 camera. For immunofluorescence microscopy, embryos were observed using a Zeiss Pascal confocal microscope. Images were processed for contrast and size in Adobe Photoshop.

### Climbing Assay

A total of 20 mated female flies were placed in a measuring cylinder. After a 1 min recovery period, the cylinder was banged firmly once on the bench, and the percentage of flies passing a 10 cm threshold within 1 min of banging was recorded. This was repeated five times each for four groups of flies from each line.

### Electron Microscopy

Whole-adult heads were removed and rinsed in 0.5% Triton X-100. The proboscis was removed to facilitate infiltration of the fix, and the heads were then fixed in 2.5% glutaraldehyde and 2% paraformaldehyde in 0.1 M phosphate buffer (pH 7.4) overnight at 4°C. Heads were then washed in 0.1 M phosphate buffer (pH 7.4), postfixed with OsO_4_, dehydrated in an ethanol series, and embedded in Polybed812. Ultrathin (75 nm) sections of the antennae were then stained with aqueous uranyl-acetate and lead citrate and examined with a Hitachi 7000 electron microscope (Electron Microscopy Research Services, Newcastle University Medical School).

### Promoter Fusions

Fragments were amplified from genomic DNA and cloned into pHStinger GFP reporter vector. Site-directed mutagenesis of these constructs was carried out using the QuikChange II Kit (Stratagene). Fox motifs were changed from RYMAAYA to RYMACGA, and X boxes were changed from GYNRCCN{1-3}RGYAAC to GYNRAAN{1-3}RGYAAC, which disrupt DNA binding in vitro by Fox and Rfx proteins, respectively (Iwama et al., 1999; Kaufmann et al., 1995). Transformants were made by microinjection into syncytial blastoderm embryos. Two to four independent transformant lines were tested in each case, and in cases of position effect-induced variability, the pattern given by the majority of lines was recorded. Confocal imaging was performed using identical nonsaturating settings for mutated and control fusion lines for direct comparison of expression levels.

### Expression and Purification of Fd3F Forkhead Domain

Total RNA was extracted from 5–21 hr embryos using RNeasy kit (QIAGEN). This RNA was reversed transcribed to produce total embryonic cDNA using ImProm-II Reverse Transcription System (Promega). The forkhead box of the gene (*fd3F^fkh^*) was amplified from the cDNA and cloned in pGEX-2T (Supplemental Experimental Procedures). The pGEX- *fd3F^fkh^* plasmid was used to transform BL21-pLysS *E. coli*. The cells were grown to OD_550_ of 0.35, and expression of Fd3F^fkh^ was then induced with 0.5 M IPTG for 2 hr at 20°C. GST-Fd3F^fkh^ was purified from the soluble cell lysate using glutathione Sepharose beads and eluted with 50 mM reduced glutathione and 0.4% deoxycholate in 250 mM Tris-HCl (pH 8).

### Gel Mobility Shift Assay

Oligonucleotides (Supplemental Experimental Procedures) were labeled with [γ^33^P]ATP (Amersham) using T4 polynucleotide kinase (New England Biolabs). Unincorporated [γ^33^P]ATP was removed using ProbeQuant G-50 microcolumns (GE Healthcare). γ^33^P-labeled duplexes were used at 0.1 nM in 20 μl reaction mixtures in binding buffer (10 mM Tris-HCl, 1 mM dithiothreitol, 1 mM EDTA, and 100 mM NaCl). The duplexes were incubated with purified Fd3F^fkh^ (12.5 μM in binding buffer) for 20 min on ice. The reaction mixture was then electrophoresed on a native 6% polyacrylamide gel in 0.5× TBE at 40 mA for 1 hr before exposure to a phosphoimager screen. For competition assays 10 nM unlabeled DNA duplex was mixed with 0.1 nM labeled DNA prior to adding Fd3F^fkh^.

### Misexpression of Fd3F

The *fd3F* ORF was amplified by RT-PCR on embryonic mRNA using a primer from the 5′ noncoding exon and the 3′ end of the annotated *fd3F* ORF (Supplemental Experimental Procedures). This yielded a fragment of 2.5 kb, which is 1 kb larger than expected from current genome annotation (http://www.flybase.org). Sequencing revealed a large ORF consisting of the coding regions of both *fd3F* and *CG32779*, which is currently annotated as a separate transcription unit within the first intron of *fd3F*. Inspection of *fd3F* orthologs from other *Drosophila* species and other insects suggests that *CG32779* is indeed part of *fd3F* (unpublished data). This ORF was therefore inserted into pUAS-attB, and transgenic flies were obtained by microinjection of syncytial blastoderm embryos.

### Electrophysiological and Biophysical Analyses

For evaluating Johnston's organ function, flies were exposed to pure tones of different intensities at the individual best frequency of their antennal sound receiver (Göpfert et al., 2005), and the resulting mechanical displacements and antennal nerve potentials were simultaneously recorded. Receiver displacements were measured with an OFV-400 Polytec laser Doppler vibrometer at the tip of the antennal arista. Nerve responses were recorded by inserting an electrolytically tapered tungsten wire between the first antennal segment and the head capsule, and an indifferent electrode placed in the thorax (Effertz et al., 2011). Sound particle velocities were measured with an Emkay NR 3158 pressure gradient microphone at the position of the fly.

## Supplemental Information

Document S1. Figure S1, Tables S1 and S2, and Supplemental Experimental Procedures
