# Supplementary material for: Forkhead Transcription Factor Fd3F Cooperates with Rfx to Regulate a Gene Expression Program for Mechanosensory Cilia Specialization
Source: Dev Cell. 2012 Jun 12;22(6):1221–33. doi: 10.1016/j.devcel.2012.05.010 (PMC3414849; doi:10.1016/j.devcel.2012.05.010)
Supplement: Document S1. Figure S1, Tables S1 and S2, and Supplemental Experimental Procedures [file mmc1.pdf]

Developmental Cell, Volume *22*

Supplemental Information

Forkhead Transcription Factor Fd3F Cooperates with Rfx to Regulate a Gene Expression Program for Mechanosensory Cilia Specialization

Fay G. Newton, Petra I. zur Lage, Somdatta Karak, Daniel J. Moore, Martin C. Göpfert, and Andrew P. Jarman

INVENTORY OF SUPPLEMENTAL MATERIAL

SUPPLEMENTAL FIGURES

Figure S1. A deletion mutation of the *fd3F* gene and additional genes that show reduced expression in *fd3F* mutant embryos. Related to Fig. 2. This shows a schematic of the deletion and the loss of fd3F expression that it causes. It also includes additional genes that could not be incorporated into Fig. 2.

SUPPLEMENTAL TABLES

Table S1. Genes whose expression is analysed in *fd3F* mutant embryos, related to Figs 3-5.

Table S2. Fox motifs and X boxes associated with *fd3F* target genes, expanding on the data shown in Fig. 6.

SUPPLEMENTAL EXPERIMENTAL PROCEDURES

Oligonucleotides used, supporting the Experimental Procedures.

Figure S1. A deletion mutation of the *fd3F* gene and additional genes that show reduced expression in *fd3F* mutant embryos, related to Fig. 2.

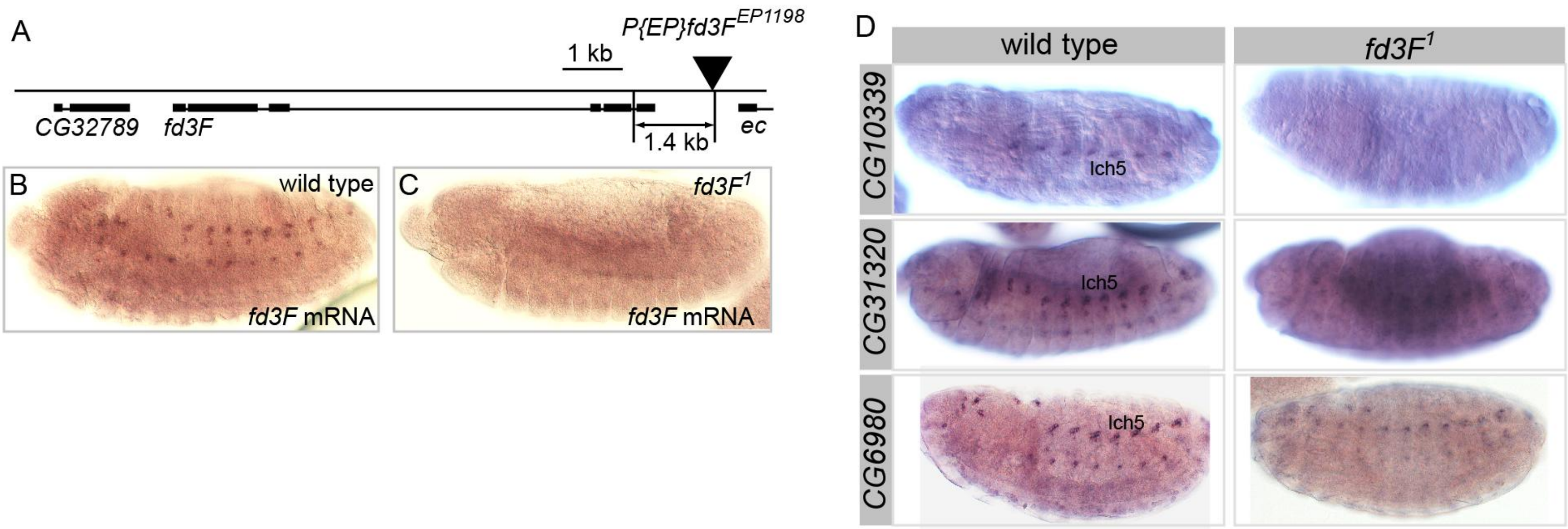

(A) Schematic of the *fd3F* gene region showing the location of the P element and the extent of the deletion caused by its excision (*fd3F*^{1} mutation). The *fd3F*^{1} mutation complements an *ec*^{1} mutation suggesting that the deletion specifically affects the *fd3F* gene. (B,C) Loss of *fd3F* mRNA from *fd3F*^{1} mutant embryo (C) compared with a wild-type embryo (B) stained in parallel. (D) Wild-type (left) and mutant (right) embryos (stages 15/16) showing mRNA expression of Ch-expressed genes (named at left).

Table S1. Gene expression in *fd3F* mutant embryos, related to Figs 2-4.
Includes genes from the initial screen (Fig. 3), axonemal motility candidates (Wickstead and Gull, 2007) (Fig. 4) and retrograde transport candidates (Ishikawa and Marshall, 2011) (Fig. 5) that are enriched in the Ch neuron transcriptome. Also included are two genes for which we could not obtain clear ISH data (nd = not determined) but are strongly predicted to be *fd3F* targets.

**Gene**	**Ch transcriptome[1]**	**Expression pattern**	**Expression in *fd3F* embryos[2]**	**Expression in *Rfx* embryos**	**X+F motif[4]**	**Orthologue[5]**	**Function**	**Foxj1 target[6]**
**(A) Genes that show reduced expression in fd3F mutant embryos**								
CG6980	33.10	Ch-specific	↓↓	↓↓	Y	*TTC12*	Unknown, TPR motifs	
CG11253	20.82	Ch-specific	↓↓	↓↓	Y	*ZMYND10*	Unknown	
btv	16.28	Ch-enriched	↓ (Ch)	↓↓	Y	*DYNC2H1*	Retrograde transport - dynein 2	
CG3769	16.11	Ch-enriched	↓↓	↓↓[3]	Y	*DYN2CLI1*	Retrograde transport - dynein 2	
CG10064	15.91	Ch-specific	↓↓	nd	Y	*WDR16*	Motility-related	X
CG10339	15.63	Ch-specific	↓	↓↓	Y		Unknown	
tektin-A	8.01	Ch-specific	↓↓	nd	Y	*TEKT4*	Axoneme stability	M, X
Oseg6	7.41	Ch-enriched	↓ (Ch)	nd	Y	*IFT144/WDR19*	Retrograde transport - IFT-A	
CG31320	7.06	Ch-specific	↓↓	↓↓	Y	*HEATR2*	Unknown	
dhc62B	6.12	Ch specific	↓↓	↓↓	Y	*DNAH3/IAD-3*	Motility - axonemal dynein, inner arm	M
CG6971	5.97	Ch-specific	↓↓	nd	Y	*DNALI1*	Motility - axonemal dynein	M, X
CG13930	5.67	Ch-specific	↓↓	nd	Y	*WDR78/IC138*	Motility - axonemal dynein	M, Z
rempA/Oseg3	4.4	Ch-enriched	↓ (Ch)	↓↓	Y	*IFT140*	Retrograde transport - IFT-A	
CG34192	4.01	Ch specific	↓↓	nd	Y	*DNALRB2*	Motility - axonemal dynein	M, X
tilB	3.87	Ch specific	↓	nd	Y	*LRRC6*	Axonemal dynein assembly	
dhc93AB	3.83	Ch-specific	↓↓	↓↓[3]	Y	*DNAH9/OADbeta*	Motility - axonemal dynein, outer arm	M, X, Z
CG9313	3.52	nd	nd	nd	Y	*DNAI1/WDR66*	Motility - axonemal dynein	M, X, Z
Oseg1	3.02	Ch-enriched	↓ (Ch)	↓↓	Y	*IFT122/WDR10*	Retrograde transport - IFT-A	
dhc16F	2.89	Ch specific	↓↓	↓↓	Y	*DNAH6/IAD-5*	Motility - axonemal dynein, inner arm	M
CG14905	2.734	Ch specific	↓	nd	N	*ODA-1* (*Chlamyd.*)	Axonemal dynein assembly	
CG5780	2.59	nd	nd	nd	Y	*IFT43*	Retrograde transport - IFT-A	
CG8800	2.51	Ch specific	↓↓	nd	Y	*DNAL1*	Motility - axonemal dynein	M, X
Oseg4	2.22	nd	↓ (RTPCR)	nd	Y	*IFT121/WDR35*	Retrograde transport - IFT-A	
nan	2.11	Ch-specific	↓↓	↓↓	Y	*TRPV4*	TRPV ion channel, active amplification gain control	
iav	-	Ch-specific	↓↓	↓↓[3]	Y	*TRPV4*	TRPV ion channel, active amplification gain control	
**(B) Genes whose expression is not altered in fd3F mutant embryos**								
dila	16.95	Ch enriched	no change	↓↓	N	*AZI1/CEP131*	Ciliary transition zone	

nompB	6.00	Ch enriched	no change	↓↓[3]	N	*IFT88*	Anterograde transport - IFT-B	
CG15161	14.22	Ch enriched	no change	↓↓[3]	N	*IFT46*	Anterograde transport - IFT-B	
CG6129	18.63	Ch-enriched	no change	↓↓[3]	Y	*CROCC*	Ciliary rootlet	
CG17564	26.67	Ch-specific	no change	nd	N	*CCDC42*	Unknown	
CG3085	19.70	Ch-specific	no change	no change[3]	N	*TEKT2*	Tektin	X
CG31291	18.39	Ch-enriched	no change	nd	N	*SDCCAG8*	Unknown	
CG13125	19.47	Ch-specific	no change	↓↓[3]	N	*LRRC48*	Unknown	
CG5359	2.75	Ch-specific	no change	no change[3]	N	*TCTEX2/DYNLT2*	Transport?	
king tubby	4.50	Pan-neural	no change	↓↓[3]	N	*TULP1*	IFT-A accessory protein?	
robo3	-	Ch specific & CNS	no change	nd	N	*ROBO*	Axon guidance receptor	
CG16789	6.62	Ch specific	no change	nd	N	*IQCA*	Unknown	M, X

[1]fold enriched in Ch cells from transcriptome data obtained from FACS sorted Ch neural precursors as described in (Cachero et al., 2011). These data are from a time point in Ch neuron development later than the data presented in this reference (4 h after precursor specification) (manuscript in preparation).

[2]↓↓ = strongly reduced/absent; ↓(Ch) = reduced in Ch neurons but not other sensory neurons.

[3]RTPCR data from *Rfx* mutant pupae (Laurençon et al., 2007)

[4]Presence of a conserved X box/Fox motif combination upstream of the translation or transcription start site. See Supplementary Table S2.

[5]Human unless otherwise stated.

[6]*Foxj1* targets from mutant transcriptome analysis in mouse (Jacquet et al., 2009), *Xenopus* (Stubbs et al., 2008) and mutant and ChIP analysis in zebrafish (Yu et al., 2008)

Table S2. Fox motifs and X boxes associated with *fd3F* target genes (related to Fig. 6). The X box sequence is shown, along with the nearest conserved Fox motif. These sequences are underlined where they conform to recognized consensus sequences for Fox proteins (RYMAAYA (Kaufmann et al., 1995)) and Rfx1 (GTNRCCN{0-3}RGYAAC (Emery et al., 1996)) Note how the X box often consists of one well-matched and one more degenerate half-site. The distance from the Fox motif to the transcription start site is indlcated if the latter is known, else the distance to the ATG is indicated. Predicted Rfx target refers to genes identified by bioinformatic analysis in Additional Data File 1 of Laurençon et al. (2007) (**L**), or Avidor-Reiss et al. (2004) (**AR**).

Name	Gene	Fox motif	Conserved	X box	Predicted Rfx target?	Conserved	Distance F to ATG	Distance F to txn	Distance X to F
nan	*CG5842*	ATCAATA	Y	GTTGCCA ATGCAAC	-	Y		+80	21
iav	*CG4536*	ACAAACA	Y	GTTACCA GGACAAC	-	Y	-76		38
btv	*CG15148*	ACAAATA	Y	GTTGCCTA GGGCAAC	L	Y	-83		11
DYN2LIC	*CG3769*	ACAAACC	Y	GTTGCTAG TAGCAAC	L	Y		+73	15
Oseg1	*CG7161*	GTAAACA	Y	AGATCCA TGGCAAC	-	Y		+12	5
Oseg6	*CG11237*	GTAAACA	Y	GCTACCA TGGAAAC	-	Y	-61		8
rempA	*CG11838*	GCCAAAA	Y	GCAACCA TGACAAC	AR	Y	-6		29
Dhc93AB	*CG3723*	GTAAACA	Y	GTTACCAC TAACAAC	L	Y	-19		46
Dhc16F	*CG7092*	ACCAACA	Y	GATGTCT GACAAC	-	Y HALF	-62		34
Dhc62B	*CG15804*	ACAAACA	Y	GTCGCCT TAGCAAC	L	Y	-87		2
WDR78 (DNAI)	*CG13930*	GTAAACA	Y	CATGGCA ACGCAAC	-	Y HALF	-46		4
DNAI1	*CG9313*	ACAAACA	Y	GAAACTGA AACAAAC	AR	Y		+83	4
DNALRB2	*CG34192*	GTCAATA	Y	GTGACCCATATGCAAC	-	Y HALF		+12	0
DNAL1	*CG8800*	ACAAACT	Y	TTCACCT TGGCAAC	AR	Y	-94		5
DNALI1	*CG6971*	GCAAACA	Y	TGCGCCTTCTAGCAAC	-	Y HALF		0	30
tilB	*CG14620*	GCCAACT	Y	AGGGCCA GGCCAAC	-	Y	-66		2
CG11253	*CG11253*	ATAAATA	N	GAATCCT AGCAAC	-	Y		-79	4
CG31320	*CG31320*	ATAAACA	Y	GTAGCCA TAGCAAC	-	Y		+4	42
CG14905	*CG14905*	GCAAACA	N	GTTACTA AGCAAC	-	N	-75		18
CG6980	*CG6980*	GTAAACA	Y	GTAACCA GGACAAC	-	N		-25	65
CG10339	*CG10339*	ATAAATA	Y	TTCGCCA TGGCAAC	-	Y HALF	-172		38
Tektin-A	*CG4767*	GCCAATA	Y	GGCACCC CAGCAAC	-	Y HALF		+53	18
CG10064	*CG10064*	GAAAACA	Y	GCAACCA AGCAAC	AR	Y		+54	40
X box (L)				RYYNYYN$_{(1-3)}$RRNRAC					
X box (AR)				GTTGCCATGGCAAC					

SUPPLEMENTAL EXPERIMENTAL PROCEDURES

Oligonucleotides

Gene	Left Primer	Right Primer	Purpose
nan	TCAATAGCCACCAATTGGACA	CCAAAACTAAGCGGAAGCAC	*In situ* hybridisation
iav	GTCGGAGAAGGAATGGATGGATGA	CAGGACATGGGCTGAAACTGT	*In situ* hybridisation
btv	AGAATCATGCGGTGATCCTC	CAAGGATTCGGATTGCAGTT	*In situ* hybridisation
Oseg6	CGTGCAAGGGAATTTACCAA	TGGGATATACAGGCGGCTAC	*In situ* hybridisation
rempA	CTGCTCTTTCCCAGCAAATC	TTCAGGACTTGCTTCCTCGT	*In situ* hybridisation
Dhc93AB	GGTGGCTGCTCTTCAGAATC	CGCTTCTCATTGGGCTTTAG	*In situ* hybridisation
Dhc16F	AACGTTCTCCTCCGCAGTAA	AGGGATTGTTTTCCCATTCC	*In situ* hybridisation
Dhc62B	CAGCAGTGACAAGGAAACGA	GGTAGCATCGATCCGTCAGT	*In situ* hybridisation
CG13930	ACGGAGGTACAGGAACATGG	ATCAGCCGGTACTTGGTCAC	*In situ* hybridisation
CG9313	GACATCCACCCAACGTATCC	TATCCTCCCACACCTTGACC	*In situ* hybridisation
CG34192	GTGGAAAAAGCATTCGACCT	AAGTAATCATGCGCGTTCTG	*In situ* hybridisation
CG8800	GTGGCTGAAACTCTGGAGGA	TGCTATGAAAGTTCGCATGG	*In situ* hybridisation
CG6971	CAAACCTGCTGAATTCCTGA	TACCTGTCTCACGAGCTTGG	*In situ* hybridisation
tilB	CCAGGAGGACATCGAGGTAA	CCTTTATGCGCTCTTCTTCG	*In situ* hybridisation
CG31320	AAGATTTGCTCGGATCTGGA	TCTTGTGATGCTGACGTAACCT	*In situ* hybridisation
CG14905	GCAGTCGATGGTAACCCAGT	GCAAAGTTCTCGTCCTCCTG	*In situ* hybridisation
Tektin-A	AGCGTATTAAGCTGCGGAAA	GTGCAGTTGATCCTGGAGGT	*In situ* hybridisation
CG5780	CAAAGTCGGCTACAAATTCCA	TGTGTGGGTGGAATCACATC	*In situ* hybridisation
fd3F	CGGGATCCTGGCTGAAATCCAAAACC	GCGAATTCTCACTCACGGTTAATCGACTC	GST expression plasmid
iav-F1	ATCATGGGTCATCGA**ACAAACA**AGCCGAGAAGGTTGT		Gel mobility shift probe
nan-F1	GCACGGAAATGTTTT**ATCAATA**GCCACCAATGGACAA		Gel mobility shift probe
iav-F1m	ATCATGGGTCATCGA**ACAACGA**AGCCGAGAAGGTTGT		Gel mobility shift probe
nan-F1m	GCACGGAAATGTTTT**ATCACGA**GCCACCAATGGACAA		Gel mobility shift probe
Fd3F-ORF	AATTCCCAATTTCCGCTCTT	GGGCTCGAGCGCTGAAACTGGAGTCTGTTG	UAS misexpression construct

SUPPLEMENTAL REFERENCES

Avidor-Reiss, T., Maer, A.M., Koundakjian, E., Polyanovsky, A., Keil, T., Subramaniam, S., and Zuker, C.S. (2004). Decoding Cilia Function: Defining Specialized Genes Required for Compartmentalized Cilia Biogenesis. Cell *117*, 527-539.

Cachero, S., Simpson, T.I., zur Lage, P.I., Ma, L., Newton, F.G., Holohan, E.E., Armstrong, J.D., and Jarman, A.P. (2011). The gene regulatory cascade linking proneural specification with differentiation in *Drosophila* sensory neurons. PLoS Biol *9*, e1000568.

Emery, P., Strubin, M., Hofmann, K., Bucher, P., Mach, B., and Reith, W. (1996). A consensus motif in the RFX DNA binding domain and binding domain mutants with altered specificity. Mol Cell Biol *16*, 4486-4494.

Ishikawa, H., and Marshall, W.F. (2011). Ciliogenesis: building the cell's antenna. Nat Rev Mol Cell Biol *12*, 222-234.

Jacquet, B.V., Salinas-Mondragon, R., Liang, H., Therit, B., Buie, J.D., Dykstra, M., Campbell, K., Ostrowski, L.E., Brody, S.L., and Ghashghaei, H.T. (2009). FoxJ1-dependent gene expression is required for differentiation of radial glia into ependymal cells and a subset of astrocytes in the postnatal brain. Development *136*, 4021-4031.

Kaufmann, E., Muller, D., and Knochel, W. (1995). DNA recognition site analysis of Xenopus winged helix proteins. J Mol Biol *248*, 239-254.

Laurençon, A., Dubruille, R., Efimenko, E., Grenier, G., Bissett, R., Cortier, E., Rolland, V., Swoboda, P., and Durand, B. (2007). Identification of novel regulatory factor X (RFX) target genes by comparative genomics in Drosophila species. Genome Biology *8*, R:195.

Stubbs, J.L., Oishi, I., Izpisua Belmonte, J.C., and Kintner, C. (2008). The forkhead protein Foxj1 specifies node-like cilia in Xenopus and zebrafish embryos. Nat Genet *40*, 1454-1460.

Wickstead, B., and Gull, K. (2007). Dyneins across eukaryotes: a comparative genomic analysis. Traffic *8*, 1708-1721.

Yu, X., Ng, C.P., Habacher, H., and Roy, S. (2008). Foxj1 transcription factors are master regulators of the motile ciliogenic program. Nat Genet *40*, 1445-1453.
